# A High Power, Frequency Tunable Colloidal Quantum Dot (CdSe/ZnS) Laser

**DOI:** 10.3390/nano7020029

**Published:** 2017-01-30

**Authors:** Saradh Prasad, Hanan Saleh AlHesseny, Mohamad S. AlSalhi, Durairaj Devaraj, Vadivel Masilamai

**Affiliations:** 1 Research Chair on laser diagnosis of cancers, College of Science, King Saud University, 11451 Riyadh, Saudi Arabia; srajendra@ksu.edu.sa (S.P.); hanan-laser@hotmail.com (H.S.A.); malsalhi@ksu.edu.sa (M.S.A.); 2 Department of Physics and Astronomy, College of Science, King Saud University, 11451 Riyadh, Saudi Arabia; 3 Department of Electrical and Electronics, College of Engineering, Kalasalingam University, Anand Nagar, Krishnankoil, Virudhunagar 626190, Tamil Nadu, India; deva230@yahoo.com

**Keywords:** quantum dots CdSe/ZnS, amplified spontaneous emission, spectral properties, broad tunable laser from 520 to 630 nm, 42.70.Hj, 85.35.Ben, 2.60.-v, 42.60.-v

## Abstract

Tunable lasers are essential for medical, engineering and basic science research studies. Most conventional solid-state lasers are capable of producing a few million laser shots, but limited to specific wavelengths, which are bulky and very expensive. Dye lasers are continuously tunable, but exhibit very poor chemical stability. As new tunable, efficient lasers are always in demand, one such laser is designed with various sized CdSe/ZnS quantum dots. They were used as a colloid in tetrahydrofuran to produce a fluorescent broadband emission from 520 nm to 630 nm. The second (532 nm) and/or third harmonic (355 nm) of the Nd:YAG laser (10 ns, 10 Hz) were used together as the pump source. In this study, different sized quantum dots were independently optically pumped to produce amplified spontaneous emission (ASE) with 4 nm to 7 nm of full width at half-maximum (FWHM), when the pump power and focusing were carefully optimized. The beam was directional with a 7 mrad divergence. Subsequently, these quantum dots were combined together, and the solution was placed in a resonator cavity to obtain a laser with a spectral width of 1 nm and tunable from 510 to 630 nm, with a conversion efficiency of about 0.1%.

## 1. Introduction

Semiconducting quantum dots have been in use for almost three decades now. However, they continue to inspire several new researches and applications every day. The commercialization of the Quantum dots (QDs) variant of ultra-high-definition (UHD) televisions opened up a vast market for quantum dots in display technology [1,2], as well as generated a great deal of public interest regarding QDs. Ultrasensitive solution-cast quantum dot photodetectors are also gaining importance [3].

Quantum dots (QD) are novel semiconducting materials which possess excitons confined to three dimensions, a typical example being the cadmium–selenide binary alloy (Cd–Se). When this type of quantum dot is prepared and placed in a suitable medium as a suspension, it disperses and fluoresces strongly in the visible region, with its fluorescence quantum efficiency comparable to that of the conventional organic laser dyes. However, they differ starkly because the absorption and re-emission in a dye are a result of the delocalization of the π electronic cloud, whereas in the case of the quantum dot, it is due to excitons. The optical properties of the type-II band engineered quantum dots [CdTe/CdSe (core/shell) and CdSe/ZnTe (core/shell) heterostructures] are unique. The spatial distribution of the carriers can be controlled within the type-II quantum dots, which enables these QDs to be used in many applications, such as photovoltaics and photoconduction devices.

These nanocrystal quantum dots present unique optical and physical properties that intermediate between molecules and bulk crystals and are strongly dependent on the particle size [3,4,5,6,7]. In these types of nanomaterials, a higher percentage of atoms are found on the surface than in the bulk: therefore, the ratio between the surface area and volume is unusually high. For instance, when the CdSe particle size drops from 10 nm to 1 nm, the ratio of the surface area to volume changes from 0.2 to 1 [8,9]. The optical absorption of the bulk CdSe typically extends up to 690 nm; however, when the particle size gets reduced to 4 nm (CdSe quantum dots), the absorption band shifts to 530 nm [10,11]. The fluorescence quantum yield of a CdSe quantum dot could be improved by encasing it in a shell, typically ZnS. This shell is termed the core/shell (CdSe/ZnS) quantum dot. The spectral properties of such CdSe/ZnS quantum dots have been studied extensively. Enhancement of the photoluminescence (PL) of the colloidal CdSe and core/shell CdSe/ZnS quantum dots has been observed when the dots are excited with photon energy higher than that of the band gap.

In an earlier report by this group, the spectral properties of the 5 nm sized bare CdSe and CdSe/ZnS core/shell quantum dots (QD) were investigated using different solvent environments with different polarities and different concentrations [12]. The photoluminescence (PL) spectra of the CdSe/ZnS core shell quantum dots revealed two bands in the non-polar solvents. In contrast, the PL spectra of the bare CdSe in non-polar solvents, showed a very strong band around 590 nm, with the complete absence of the primary wavelength band at 420 nm [13,14].

Meanwhile, a few quantum dots have been induced to produce a laser employing different material configuration and pumping techniques. The most important example is the InAs system emitting a laser at 1.5 µm under electrical excitation, very similar to the conventional semiconductor lasers. A current injection of 40 mA was required to produce a laser of 10 mW at 50 °C. Another example was the InP quantum dot, with a laser at 661 nm with a threshold current density of 300 A/cm^2^. Besides these, a laser by optical excitation has also been occasionally tried. The nearest to our line of research was the CdSe/ZnS colloidal quantum dot having a 5 nm mean diameter, deposited and trapped as a thin film in a glass grove and pumped by the Nd:YAG laser; this quantum dot lased at 600 nm due to the distributed feedback from the groves [14].

In a few other reports, in the heterogeneous wavelength-tunable laser diode constructed using quantum dot (QD) and silicon photonics technology, the authors took advantage of the large optical gains in the 1000–1500 nm wavelength in the infrared (IR) region and used a scalable platform to produce complex integrated photonic devices [15,16,17].

Anantathanasar et al. demonstrated amplified spontaneous emission (ASE) from the PbSe nanocrystals (NCs) with emission tunable in the near-infrared (NIR) for the first time. Their work showed that despite the eight-fold degeneracy of the lowest quantized states and fast non-radiative Auger recombination, the optical gain parameters of PbSe NCs were very high [18]. It is well known that the higher the wavelength of emission, the higher will be the optical gain because optical gain depends on the fourth power of the wavelength (λ^4^). Therefore, it is much harder to achieve laser emission in the ultraviolet (UV) region than in the visible or IR region. In spite of such difficulty, a few research groups have achieved laser action in a visible spectrum; some important works are given below.

One such important result was recorded by Bawendi et al. They reported the optical amplification and lasing dynamics involved in nanocrystal quantum dots. Excited state dynamics revealed that the non-radiative auger effect dominated the recombination process and stimulated emission was achieved only in the closely-packed solids of the nanocrystals. They also researched tunable lasers by varying the nanocrystal sizes [19]. In 2004, Caruge et al. indicated that the nonlinear excitation of the CdSe/ZnS core/shell nanocrystals, embedded in the high volume fraction in a TiO_2_ matrix, produced amplified spontaneous emission (ASE) from the multi-excitonic band [14].

The ASE is sometimes termed as a mirror-less laser emission arising out of a single pass optical gain. When the pump source (e.g., the Nd:YAG laser or excimer laser) excites the medium (e.g., dye solution) it produces high population inversion, and spontaneous emission generated at any point of the inverted medium, gets amplified exponentially on either side to produce ASE with a high degree of collimation (5 milliradian) and spectral narrowing (5–7 nm) [20].

In 2012, Dang et al. showed that the colloidal quantum dots in the thin film (CdSe/ZnCdS core/shell) exhibited high quantum yield and optical gain. They achieved amplified spontaneous emission (ASE) with low pump energy density of 90 µJ·cm^−2^. When the pump power increased above the threshold, the spectral peaks at 625 nm narrowed from 28 nm to 7 nm full width at half-maximum (FWHM). The following were significant factors in their work: (a) a 100 fs, 400 nm laser pump source was used (second harmonic of Ti: Sapphire); (b) 100 kHz pulse repetition rate and (c) 148 mg/mL concentration [21].

In our recent work, the CdSe/ZnS quantum dot was shown to produce a weak ASE with efficiency around 0.05% [22]. As the tunability was only about 20 nm, we continued in the same line of work to improve the tunability and efficiency by employing multiple QDs and utilized appropriate conjugated polymers (CPs) to improve the performance via energy transfer.

In the present study, we report the tunable laser from the different sized CdSe/ZnS quantum dots (CdSe as core and ZnS as shell) placed as a suspension in THF. A frequency tripled Nd:YAG laser was used to pump the CdSe/ZnS quantum dots to produce super-irradiant lasing from different wavelengths with 0.1% conversion efficiency. A mixture of all the quantum dots along with the PFO and MEH-PPV produced an almost flat efficiency photoluminescence, which when placed under a resonator cavity produced a laser with full width half-maximum (FWHM = 1 nm) having a 120 nm tunable range (from 520 to 630 nm). To the best of our knowledge, this could be the first report on a high-power, wide-range, tunable laser from a quantum dot conjugated-polymer mixture. This new tunable laser engineering could open up new opportunities for the quantum dots as tunable laser materials comparable to conventional laser dyes or the more recent conjugated polymers.

## 2. Results and Discussion

### 2.1. Spectral Properties

**Figure 1:**
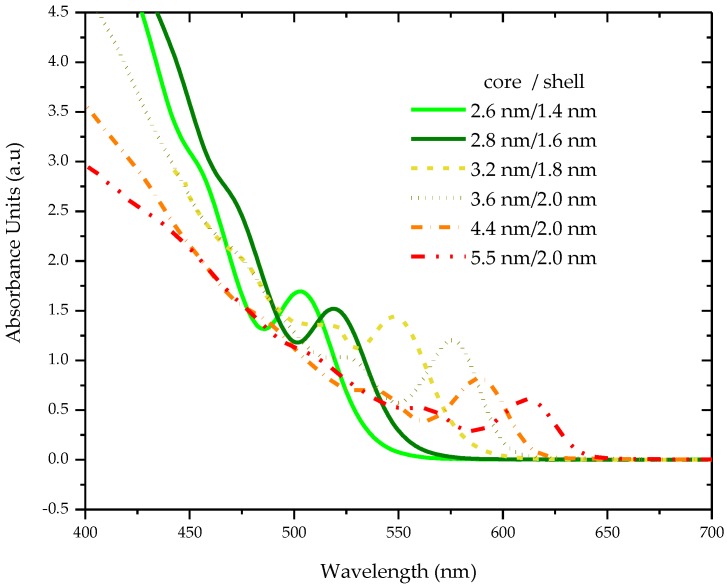
The absorption spectra for different sized quantum dots at a concentration of 2 mg in 5 mL of THF.

The absorption spectra were recorded for a wide concentration range of different sized CdSe/ZnS quantum dots (2.6 to 5.5 nm size) in tetrahydrofuran (THF). The concentrations ranged from 1 to 50 mg in 5 mL of THF. The absorption spectra for all these concentrations revealed two peaks: one primary with a Strokes shift Δλ ≈ 20 nm (from fluorescence peak) in longer wavelengths and a shoulder with increasing absorbance in shorter wavelengths and the UV region; the band intensities of both increased monotonically at the higher concentration, with no changes in the spectral features; such spectra were indicative of the absence of any aggregation [19]. Figure 1 shows the absorption spectra for different sizes of quantum dots at a concentration of 2 mg in 5 mL of THF. Figure 1 reveals the spectral profile of the absorption spectra from which two important observations are made: (a) absorbance increases with the decrease in the QDs; (b) the absorbance peak gets blue shifted as the quantum dots decrease in size. The absorbance increases with the reduction in the size of the quantum dots and the peaks are tabulated in Table 1.

**Figure 2:**
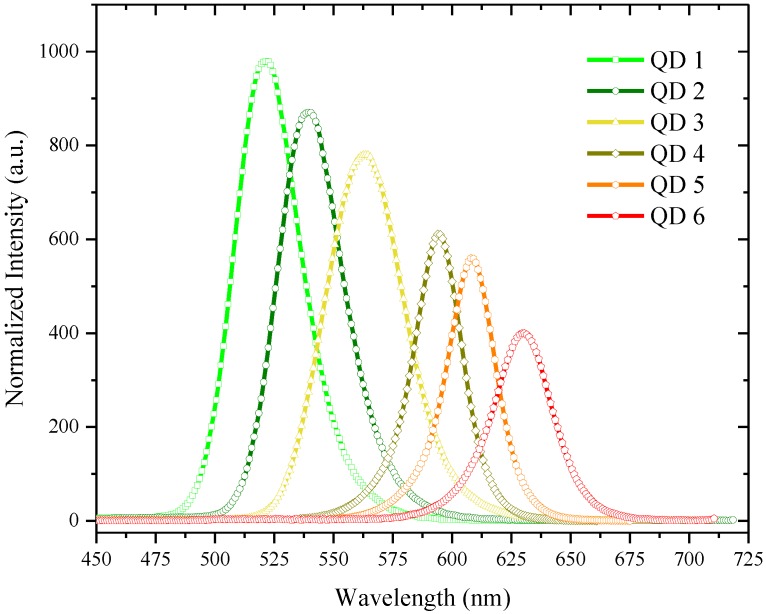
The photoluminescence (PL) for the different sized quantum dots at a concentration of 2 mg in 5 mL of THF.

Each quantum dot was dissolved in the THF over a wide concentration range (1 to 50 mg in 5 mL) and their photoluminescence (PL) revealed that the intensity increased with the rise in concentration for a certain range and then the intensity declined upon further increase in concentration. Figure 2 presents the PL spectra of the different sized dots of CdSe/ZnS in THF at the same concentration used for recording the absorption spectra (2 mg in 5 mL). PL spectra showed a peak and a band with a full width at half-maximum (FWHM) ≈ 27 nm. Photoluminescence also shows that intensity decreases with an increase in quantum dot size.

The fluorescence quantum yield was measured using the following equation [23].

$$\Phi_{F}(S)=\Phi_{F}(R)\;\left[\frac{\int I_{S}(\bar{\nu })d\nu }{\int I_{R}(\bar{\nu })d\nu }\times \frac{A_{R}}{A_{S}}\times \frac{n_{S}^{2}}{n_{R}^{2}}\right]$$

where the S and *R* indices refer to the sample and reference, respectively, and the integral over *I* represents the area under the fluorescence spectrum. “*A*” and “*n*” are the optical density and refractive index of the solvents, respectively. A_S_ this QDs displayed an emission band from 520 to 630 nm, the most appropriate fluorescence standard was Rhodamine 6G (R6G) in ethanol. By comparing the absorption with the fluorescence spectra of the standard (R6G) and sample (QDs), the fluorescence yield was found to be around 60%. A detailed report on quantum yield calculation of QDs had been provided earlier by the same group [11,12].

### 2.2. Laser Induced Fluorescence (LIF) and ASE

**Figure 3:**
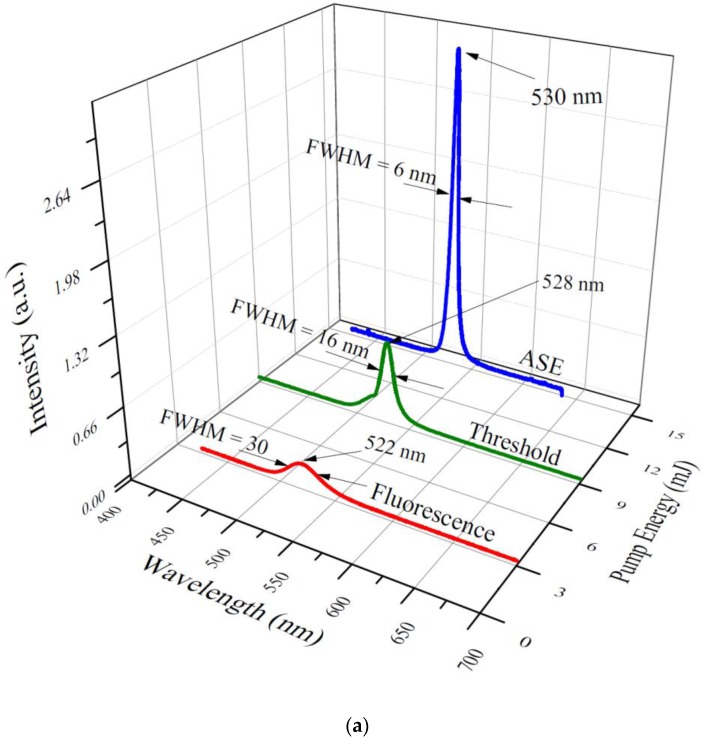

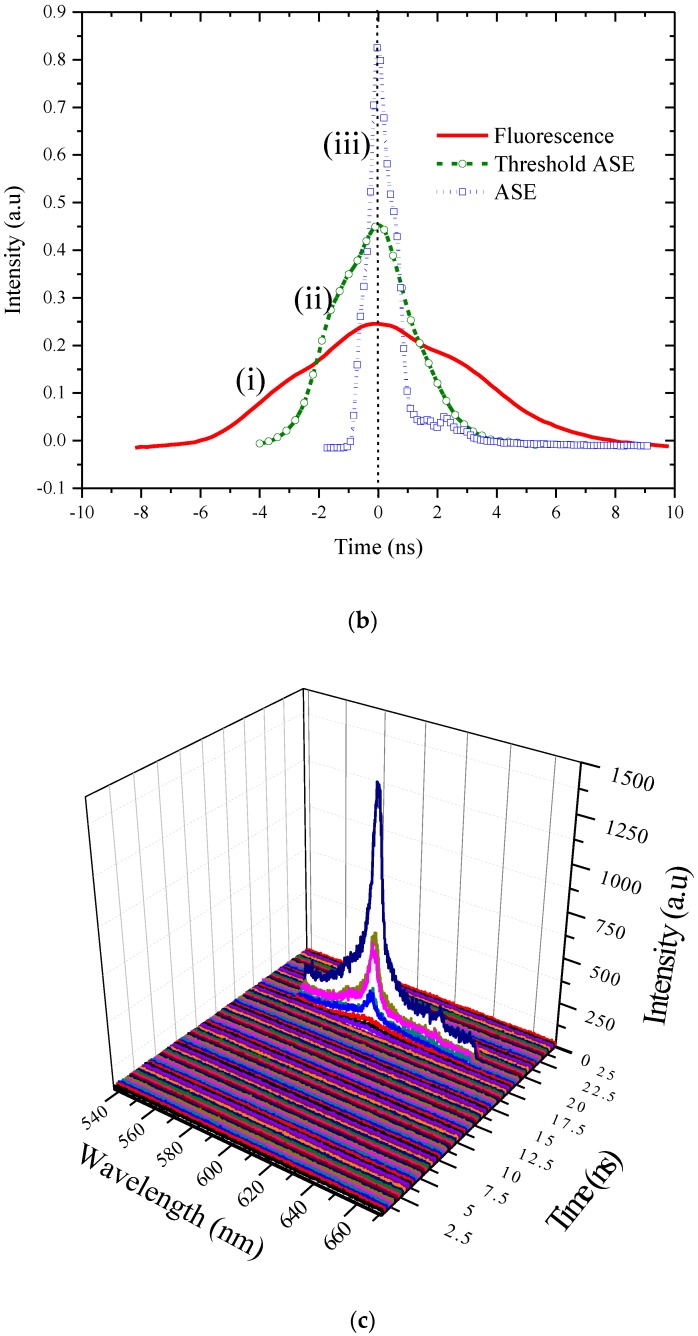
(**a**) The spectral profile of the quantum dots (QD 1) at a concentration of 2 mg in 5 mL of THF; (**b**) The temporal profile of the quantum dots (QD 1) at a concentration of 20 mg in 5 mL of THF; (**c**) Depicts the three dimensional (3D) temporal and spectral profile for another quantum dot (QDs 5, 609 nm) at 24 mg in 5 mL of THF. To differentiate one time frame from other, difference color line are used, each frame represent 0.25 ns.

The CdSe/ZnS (520 nm, QDs 1) dispersed in THF at 20 mg/ 5mL concentration was transversely excited with a UV laser at 355 nm and the pump energy was maintained at 3 mJ. The laser-induced fluorescence (LIF) was obtained, as shown in Figure 3a. It was evident that the band at 523 nm had a full width at half-maximum (FWHM) of 30 nm. When the pump energy was increased to 9 mJ, the LIF rose in intensity with a corresponding narrowing in the spectral width to FWHM of 16 nm. At 15 mJ pump energy, it became an amplified spontaneous emission (ASE) arising from the both sides of the cuvette. The ASE occurred with a peak at 530 nm showing a spectral narrowing (Δλ = 6 nm) and directionality of 8 milliradian. The ASE was a collimated small spot, 2 mm in diameter, with energy of 15 μJ, and efficiency of 0.1%.

In order to monitor the temporal profiles of the LIF and ASE from the green quantum dot laser, the emissions were put into an ultrafast Si photodetector (UPD) connected to an oscilloscope, the trigger signal being acquired from the Q switch out of the pump laser. Figure 3b (i) shows the temporal profile of the fluorescence (LIF) at 520 nm. This possessed a pulse with (FWHM) of 9 ns, having an almost smooth bell-shaped profile. Figure 3b (ii) gives the pulse shape of the LIF threshold, showing a pulse width of only 4 ns, with a rise-time of 1 ns and a fall-time of 3 ns. In Figure 3b, (iii) the ASE temporal profile is seen with 900 ps FWHM showing a very sharp rise-time (300 ps) and a longer fall-time (600 ps). This was obtained for a solution concentration of 20 mg in 5 mL of THF and pulse energy, as discussed above.

It must be noted that the LIF profile does not represent the true excited life-time of the state of the quantum dots, because the sample was excited by a laser pulse of 10 ns pulse duration and the excited state life-time of the quantum dots was around 0.9 ns. Nevertheless, it presents evidence for the temporal narrowing, a condition often exhibited while optical gain is achieved in the pulsed laser. Figure 3c shows the three dimensional (3D) temporal and spectral profile for the ASE of the quantum dots (QDs 5, 609 nm), revealing that it takes 17.5 ns time to build up the population inversion and the ASE exists only for a brief period ~2 ns. The spectra were taken for a concentration of 20 mg in 5 mL.

The energy output of ASE was measured from both sides of the cuvette. When QD1 was pumped with an energy of 15 mJ, the measured output energy of ASE was 15 µJ, corresponding to an efficiency of 0.1%. The output energy rose sharply for the pump energy above the threshold as shown in Supplementary Materials Figure S1. This was the best ASE efficiency; all Quantum dots work with less and less efficiency. For other QDs, the ASE was measured and given in Table 2.

**Figure 4:**
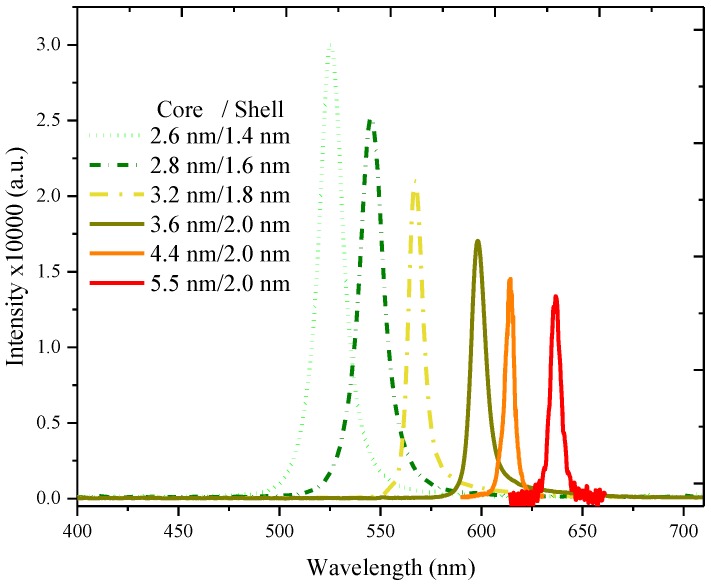
The amplified spontaneous emission (ASE) spectra profile of all quantum dots (QD 1 to QD 6) for a concentration ranging between 20 and 30 mg in 5 mL THF.

Similar results were obtained using other QDs, in which the ASE efficiency of the QDs decreased with the increase in the size of the quantum dots. The QDs 1 was the best, with the highest efficiency and QDs 6 was the least efficient of all the QDs, as shown in Figure 4.

The following facts are significant: (a) the ASE could be obtained by transverse excitation as in the case of conventional laser dyes, such as fluorescein or coumarin or the more recent conjugated polymers MEH-PPV; (b) the optical gains of these QDs are comparable to the conventional laser dyes such as Rhodamine B or a conjugated polymer such as MEH-PPV; also, the quantum yields of the fluorescence are comparable [12].

**Figure 5:**
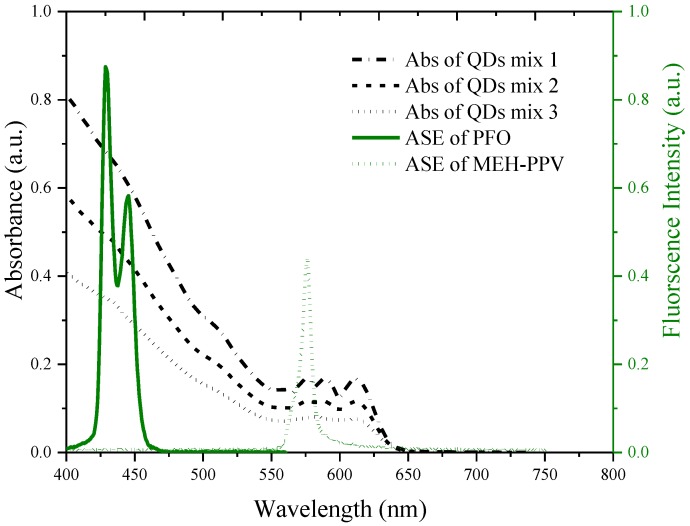
The energy transfer cross-section between QDs and poly (9, 9-dioctylfluorenyl-2,7-diyl) (PFO) and poly [(9,9-dioctylfluorenyl-2,7-diyl (PFO) and poly [2-methoxy-5-(2-ethylhexyloxy)-1,4-phenylenevinylene] (MEH-PPV).

**Figure 6:**
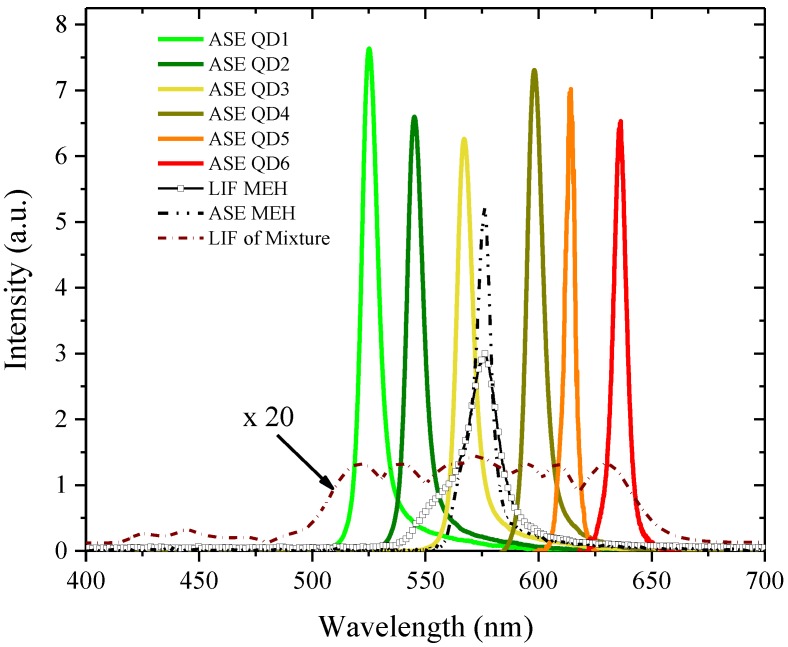
The energy transfer from PFO and MEH-PPV to QDs; here, the ASE efficiency was improved at least three-fold from the prior condition to the energy transfer. The performance of QD 4, QD 5, and QDs 6 increased notably after the addition of MEH-PPV.

In order to improve the lasing characteristic of the QD, one may need the energy transfer process. The conjugated-polymer, poly(9, 9-dioctylfluorenyl-2,7-diyl) (PFO) with the absorption peak around the 355 and ASE peaks, one at 420 nm and the other at 440 nm, is a very good candidate for the energy transfer to the QDs. Figure 5 shows the absorption spectra of the mixed solution of QDs and the ASE spectra of the PFO. The details of the energy transfer mechanism will be described in future reports. When the PFO and QDs concentrations are properly adjusted, the energy transfer occurs, improving the efficiency of the laser action. For example, when the PFO of 10 mM (2 mL) concentration was added to the QDs mixed solution (QD1 = 5 mg/mL, QD2 = 7 mg/mL, QD3 = 6 mg/mL, QD4 = 7.5 mg/mg, QD5 = 8.0 mg/mL, and QDs = 10 mg/mL), a two-fold improvement of the ASE of the QDs mixture was seen, as shown in Figure 6.

Without MEH-PPV, we were able to observe the laser from 435 nm to 630 nm with a gap/low output efficiency at 560 nm (see Figure S2). In order to improve the efficiency of output power around 560 nm, MEH-PPV was added in a small quantity. This absorbs a substantial portion of pump energy and transfers energy to QD4, QD5 and QD6, so that laser action could be achieved with almost flat efficiency from 520 nm to 630 nm.

The ASE efficiency dips around 570 nm. To compensate for this efficiency decrease, 2 mL of the MEH-PPV solution, 100 μM in concentration, was added to the above mixture. The MEH-PPV increased the laser action efficiency around 570 nm, as well as enhanced the overall efficiency. It especially improved the efficiency of the quantum dots which emitted the longer wavelength (QD4, QD5 and QD6), as shown in Figure 6. Using the trial and error method, a few other combinations of QDs, PFO and MEH-PPV were identified and similar performance was achievable, but the combination mentioned above is one of the best.

**Figure 7:**
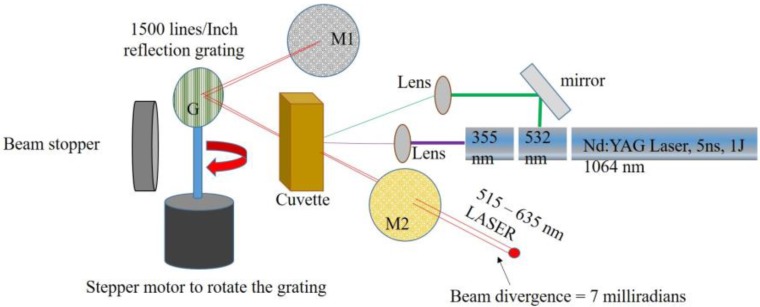
The laser set-up.

Figure 7 shows the cavity set-up; for QD 1 and QD2, the excitation source was 355 nm (third harmonic) and for the other QDs, both 355 nm and 532 nm (second harmonic) were simultaneously used as the pump source. The cavity contained a 100% reflecting mirror M1, and 60 reflecting output mirrors M2 with a reflection grating G for wavelength selection. (Edmund optics, York, UK).

**Figure 8:**
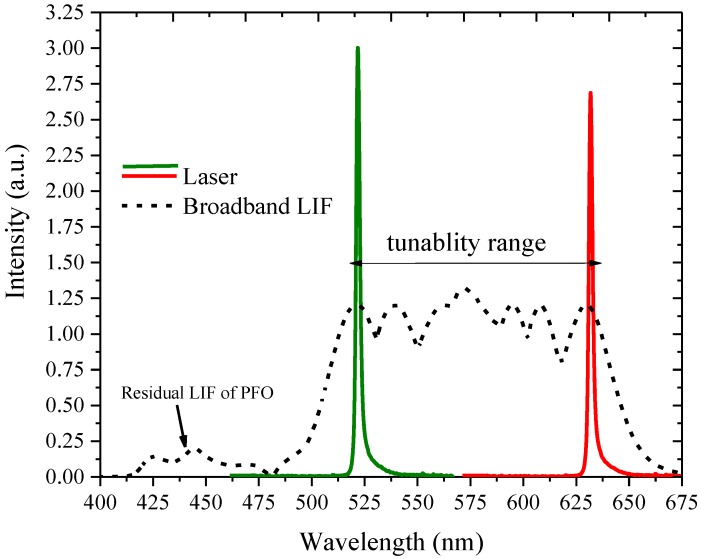
Narrow laser spectra and Laser Induced Fluorescence (LIF) of the solution.

Figure 8 shows the broadband LIF with an almost flat-top fluorescence intensity spectral profile from 510 to 630 nm for the sample without the feedback, and when placed under the cavity, the laser which emanated had a 1 nm spectral width and was 0.1% efficient. For gross tuning, the concentration was changed and for fine tuning the grating was used.

## 3. Materials and Methods

The CdSe/ZnS quantum dots were prepared adhering to the procedures reported earlier: TOPO (trioctylphosphine oxide) covered CdSe nanocrystals were produced using the standard published methods [24]. The CdSe QDs were purified applying the multiple precipitation/redissolution method. Then, a few ZnS monolayers were deposited around the CdSe cores by using the successive ion layer adhesion and reaction (SILAR) method [22]. To improve the ZnS shell growth on the CdSe core by SILAR using elemental sulfur and zinc oxide as the precursors, a stock solution of 0.1 M concentration was prepared for ZnO, oleic acid and ODE (1-octadecene) used for the Zn coating. To coat the sulfur, a stock solution of S with ODE was used. The purified CdSe QDs were added to a reaction flask containing hexadecylamine (HDA) and ODE, where the Zn and S stock solutions were added under argon flow to produce the ZnS shell. To optimize the shell growth, the reaction temperature was controlled between 200 °C and 240 °C.

**Figure 9:**
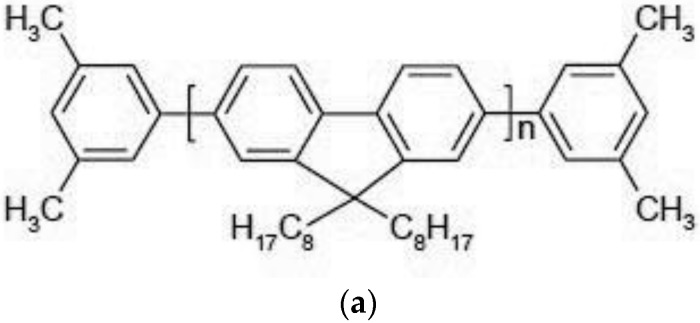

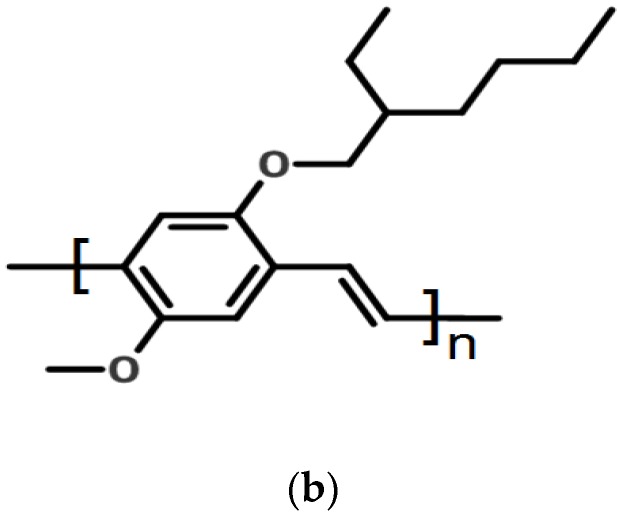
The molecular structures of (**a**) PFO and (**b**) MEH-PPV.

The conjugated-polymers (CPs), poly [(9,9-dioctylfluorenyl-2,7-diyl (PFO) and poly [2-methoxy-5-(2-ethylhexyloxy)-1,4-phenylenevinylene] (MEH-PPV) were purchased from American Dye Source, Canada and Ossila, UK respectively and were used as received. PFO and MEH-PPV are macromolecules with molecular mass of 120,000 and 100,000, respectively. Thin layer chromatography (TLC) examination revealed that the sample purity for each was above 98%. The molecular structure of the conjugated polymer PFO and MEH-PPV are shown in Figure 9; PFO was dissolved in tetrahydrofuran (THF).

The absorption and fluorescence spectra of the QDs, MEH-PPV and PFO in THF were recorded for different concentrations. The spectra of the solutions were measured using a small quartz cuvette with the dimensions 1 × 1 × 4 cm with a 1 cm optical path length.

The absorption spectra were recorded using a Perkin Elmer lambda 950 spectrophotometer over a 330 to 550 mm range and the fluorescence spectra were recorded using a Perkin Elmer LS 55 spectrofluorometer in the 400 to 500 nm range, at room temperature. The excitation wavelength was 355 nm.

The third harmonic (355 nm) of the Nd:YAG laser (Brilliant B of Quantel), with a 5 ns pulse width, was utilized as the excitation source. The UV laser was focused using a quartz cylindrical lens of 5 cm focal length. This was used to perform a transverse excitation of the samples (QDs and CPs solutions) which were taken in a quartz cuvette kept tilted to avoid Fresnel feedback, (See references [25,26] for more details). At optimum values of the pump power and PFO concentration, amplified spontaneous emission could be achieved, which emerged as a light cone, with 5 milliradian divergence. This was collected by a 1-mm entrance slit of the spectrograph connected to an Electron Multiplying Charge Coupled Device (emCCD) camera, to obtain the spectral features of the amplified spontaneous emission (ASE). An ultrafast Si photodetector (UPD) (ALPHALAS GmbH: P/N:UPD-200-UP, Rise-time: 120ps) and sampling oscilloscope (Textronix: DPO4104B-L, Digital Phosphor: 1GHz, 5 GS/s, four channels) with a combined time resolution of 200 ps, was used to monitor the temporal properties of the ASE pulse from the QDs [27].

## 4. Conclusions

In this study, simultaneous excitation of six QDs was done, using the second and third harmonics of the Nd:YAG laser as the optical pump source. With the appropriate selection of pump energy and concentration, each quantum dot could be induced to generate ASE individually from 530 nm to 630 nm; however, in a mixture of all of the six QDs mentioned above taken together, and with the energy transfer from two conjugated polymers, viz. MEH-PPV and PFO, it was possible to get ASE tunable from 520 to 630 nm with almost the same efficiency with a spectral width of 7 nm (FWHM) in the ASE mode and of 1 nm in the cavity mode. The overall efficiency was 0.1%, which indicated pulse energy of 15 μJ and peak power of 16.7 kW.

## Figures and Tables

**Table 1:** Details about Quantum dots size, absorption emission and Strokes shift.

Quantum Dot Name (Identifier)	Size of Quantum Dot		Absorption Peak (nm) λ_abs_	Emission Peak (nm) λ_flu_	Strokes Shift
	Core (nm)	Shell(nm)			Δλ = λ_flu_ – λ_abs_
QDs 1	2.6	1.4	501	521	20
QDs 2	2.8	1.6	519	540	21
QDs 3	3.2	1.8	541	563	22
QDs 4	3.6	2.0	575	595	20
QDs 5	4.4	2.0	590	609	19
QDs 6	5.5	2.0	611	630	19

**Table 2:** Measured input, output and efficiency.

	Input (mJ)	Output (µJ)	Efficiency (%)
QD1	15	0.015	0.1
QD2	15	0.0147	0.098
QD3	15	0.01455	0.097
QD4	15	0.0123	0.082
QD5	15	0.0105	0.07
QD6	15	0.009	0.06

## Supplementary Materials

The following are available online at http://www.mdpi.com/2079-4991/7/2/29/s1.
