# Supplementary material for: A High Power, Frequency Tunable Colloidal Quantum Dot (CdSe/ZnS) Laser
Source: Nanomaterials (Basel). 2017 Jan 30;7(2):29. doi: 10.3390/nano7020029 (PMC5333014; doi:10.3390/nano7020029)
Supplement: Supplementary file 1 [file nanomaterials-07-00029-s001.pdf]

Supplementary Materials: A High Power, Frequency Tunable Colloidal Quantum Dot (CdSe/ZnS) Laser

Saradh Prasad, Hanan Saleh AlHesseny, Mohamad S. AlSalhi, Durairaj Devaraj and Vadivel Masilamai

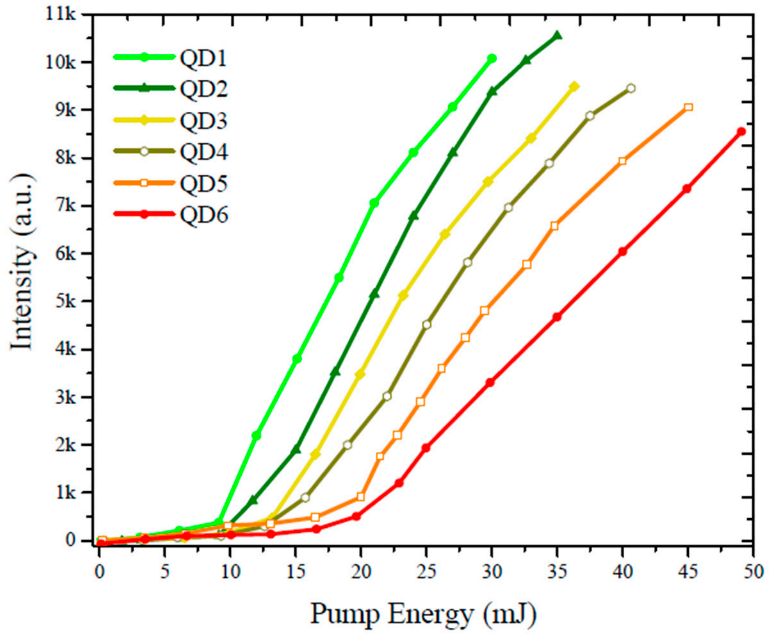

Figure S1. Input pump energy (mJ) vs Output Intensity (a.u.)

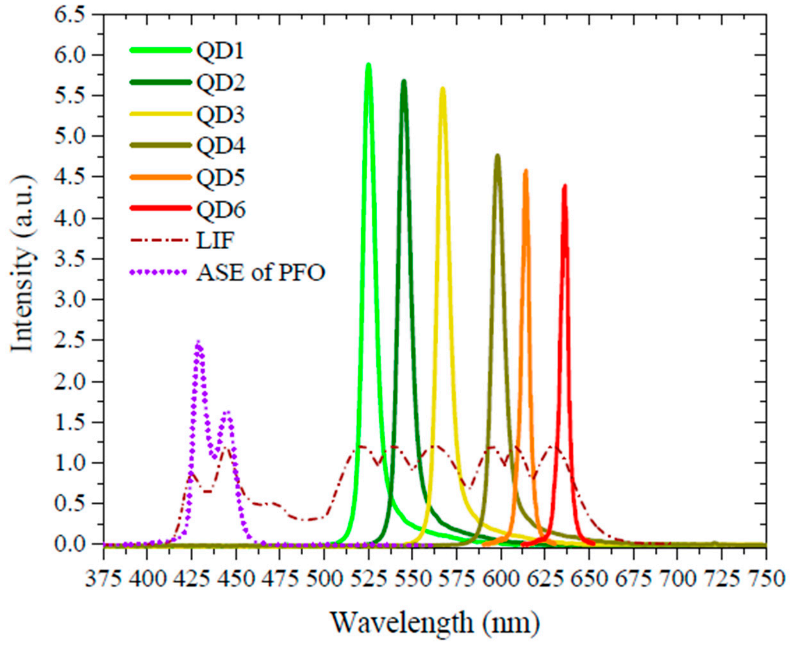

Figure S2. The energy transfer from PFO to QDs; here, the ASE efficiency was improved from the condition prior to the energy transfer. Note, the performance of QD 4 QD 5 and QDs 6 is low before the addition of MEH-PPV.
